# A New Model of Cuprizone-Mediated Demyelination/Remyelination

**DOI:** 10.1177/1759091414551955

**Published:** 2014-09-26

**Authors:** Hilary H. Sachs, Kathryn K. Bercury, Daniela C. Popescu, S. Priya Narayanan, Wendy B. Macklin

**Affiliations:** 1 Department of Cell and Developmental Biology, University of Colorado School of Medicine, Aurora, CO, USA; 2 Department of Neurosciences, Lerner Research Institute, Cleveland Clinic, Cleveland, OH, USA

**Keywords:** Akt, cuprizone, demyelination, mTOR, oligodendrocyte, rapamycin

## Abstract

In the central nervous system, demyelinating diseases, such as multiple sclerosis, result in devastating long-term neurologic damage, in part because of the lack of effective remyelination in the adult human brain. One model used to understand the mechanisms regulating remyelination is cuprizone-induced demyelination, which allows investigation of remyelination mechanisms in adult animals following toxin-induced demyelination. Unfortunately, the degree of demyelination in the cuprizone model can vary, which complicates understanding the process of remyelination. Previous work in our laboratory demonstrated that the Akt/mTOR pathway regulates active myelination. When given to young postnatal mice, the mTOR inhibitor, rapamycin, inhibits active myelination. In the current study, the cuprizone model was modified by the addition of rapamycin during cuprizone exposure. When administered together, cuprizone and rapamycin produced more complete demyelination and provided a longer time frame over which to investigate remyelination than treatment with cuprizone alone. The consistency in demyelination will allow a better understanding of the mechanisms initiating remyelination. Furthermore, the slower rate of remyelination provides a longer window of time in which to investigate the diverse contributing factors that regulate remyelination. This new model of cuprizone-induced demyelination could potentially aid in identification of new therapeutic targets to enhance remyelination in demyelinating diseases.

## Introduction

Myelination of the nervous system is essential for facilitating rapid nerve conduction and providing trophic support for axons. Demyelination leads to serious neurologic consequences as in diseases such as multiple sclerosis. Identifying remyelination therapies is therefore important, and a number of demyelination models have been used to define stages of demyelination and remyelination, with the goal of identifying potential remyelination therapeutics. Many of these models focus on experimental autoimmune encephalomyelitis (EAE), an immune-mediated demyelination that is induced by myelin antigen injection. Although this model has contributed to understanding the immune component of autoimmune attacks on myelin, it is somewhat harder to dissect out elements of demyelination and remyelination that are essential central nervous system (CNS) responses. Two major models have been developed to investigate demyelination independently of immune attack: the lysolecithin demyelination model and the cuprizone demyelination model. Cuprizone [oxalic acid bis(cyclohexylidene hydrazide)], a copper chelator, induces demyelination in the corpus callosum, hippocampus, and some other white matter regions of the rodent CNS. Its underlying mechanism of demyelination is not well understood, but cuprizone has been used to induce CNS demyelination for many decades. It has been noted that mouse strain, age, or gender impact the degree of demyelination (see Kipp et al., 2009, for a review). Furthermore, some variability results from an extensive attempt to remyelinate the CNS, which starts early, even before cuprizone exposure terminates (Matsushima and Morell, 2001). Thus, while cuprizone intoxication is inducing demyelination, oligodendrocyte progenitor cells (OPCs) are proliferating and differentiating to remyelinate axons. For studies focused on understanding the regulation of demyelination and remyelination, this mixture of cell responses is a problem.

The current studies developed out of findings in this laboratory, which demonstrated that a major signaling pathway regulating myelination is the Akt/mTOR pathway (Flores et al., 2008; Narayanan et al., 2009). We hypothesized that to study exclusively demyelination and then study a well-defined remyelination period after removal of cuprizone, it was important to eliminate the underlying attempt of OPCs to remyelinate axons. Our earlier data demonstrated that intraperitoneal injection of rapamycin, a potent inhibitor of mTOR activity during active myelination, reduces myelin production. We reasoned that the underlying attempt to remyelinate during cuprizone treatment could be eliminated by inhibition of mTOR. Thus, we treated mice with a combination of rapamycin and cuprizone to prevent remyelination during the demyelination period and have established a more effective model in which to study the regulation of remyelination. This model will have significant relevance for understanding the regulation of remyelination and for testing potential therapeutics to enhance remyelination. It provides greater demyelination and a longer remyelination period in which to quantify the impact of remyelination therapeutics.

## Materials and Methods

### Animals and Induction of Demyelination

The Institutional Animal Care and Use Committee at the University of Colorado School of Medicine and the Cleveland Clinic Lerner Research Institute approved all experimental procedures. Eight-week-old male C56BL/6 J mice (a total of 132) were obtained from the Jackson Laboratory (Bar Harbor, MN). Food and water were available ad libitum, and mice were weighed weekly. Demyelination was induced by 0.3% cuprizone [bis(cyclohexanone)oxaldihydrazone; Sigma-Aldrich, Inc., St. Louis, MO] or 0.3% cuprizone in combination with rapamycin (LC Laboratories, Woburn, MA). Briefly, one group underwent demyelination by feeding the mice a diet containing 0.3% cuprizone mixed into Teklad Global Rodent Diet (Harlan Laboratories, Inc., Indianapolis, IN). These mice received intraperitoneal injections 5 days a week with a vehicle solution consisting of a final concentration of 5% polyethylene glycol 400 (PEG 400), 5% Tween 80 (both from Sigma), and 5% ethanol (cuprizone/vehicle, Cup mice, *n* = 43). Control mice for this group received regular Global Rodent Chow and the vehicle injections (vehicle, Veh mice, *n* = 25). A third group of mice was fed the 0.3% cuprizone diet and, in addition, received injections of rapamycin (10 mg/kg; Sarbassov et al., 2006; Narayanan et al., 2009) dissolved in the vehicle (cuprizone/rapamycin, CupR mice, *n* = 42). Control mice for this group received rodent chow in combination with rapamycin injections (rapamycin, Rapa mice, *n* = 32). The cuprizone chow was changed every other day. After 6 weeks of treatment, all cuprizone feedings and all injections were discontinued. The mice were either perfused for immunohistochemical analysis or electron microscopy (EM; as described later) after 4 or 6 weeks, or they were returned to a regular diet and allowed to recover for 1, 3, 5, or 7 weeks prior to tissue analysis. Each group consisted of at least four mice for immunohistochemistry and two mice for EM.

### Tissue Preparation for Immunohistochemistry

Mice were perfused transcardially with phosphate-buffered saline (PBS) followed by cold 4% paraformaldehyde in PBS. Brains were removed, postfixed overnight in 4% paraformaldehyde, and transferred to cryoprotection solution (20% glycerol in 0.1 M Sorensen’s Buffer, pH 7.6). Coronal frozen sections (30 µm, free floating) were cut on a Leica sliding microtome (SM200R; Leica Microsystems, Inc., Buffalo Grove, IL), transferred to cryostorage solution (30% ethylene glycol, 30% sucrose, 1% PVP-40 in 0.2 M Sorensen's Buffer), and stored at −20°C.

### Myelin Oligodendrocyte Glycoprotein Immunohistochemistry and Image Analysis

Free-floating brain sections were washed (all washes were in PBS), incubated in 10% Triton X-100 with 1% H_2_O_2_ (Sigma) for 30 min, washed, and blocked with 5% normal donkey serum (NDS). Myelin was detected by incubating sections overnight at 4°C with myelin oligodendrocyte glycoprotein (MOG) antibody (rabbit, 1:2,000; Abcam, Cambridge, MA) in 2% NDS. Sections were washed, incubated for 1 hr with a biotinylated donkey anti-rabbit secondary antibody (1:1,000; Jackson ImmunoResearch Laboratories, Inc., West Grove, PA) followed by incubation with an avidin-biotin complex (ABC reagent, 1:1,000; Vector Laboratories, Burlingame, CA). Sections were washed and incubated in diaminobenzidine (DAB kit, Vector Laboratories), and the myelin labeling was amplified with 0.08% osmium tetroxide (OsO_4_) for 20 s (Trapp et al., 1997). The sections were transferred to 60% glycerol, mounted onto glass slides, coverslipped with 100% glycerol, and sealed with nail polish. Images of MOG immunoreactivity were acquired with a Leica DMR microscope and a 10 × objective (NA 0.25) with a DFC290HD digital camera using Leica Application Suite (LAS) software (Leica Microsystems). Flat-field correction was applied to equalize the image intensity across the field prior to capture. To quantify the amount of MOG immunoreactivity in the corpus callosum, pixel intensities values were calculated using ImageJ software (http://rsb.info.nih.gov/ij/) from exported TIFF files. Due to the variability of labeling that can occur with DAB staining, all images used for quantification were compared with their respective control tissues, labeled at the same time, and imaged under identical conditions. The area of interest for analysis included the corpus callosum between the midline and below the apex of the cingulum. Normalization was carried out by thresholding to exclude values that were below MOG reactivity. The density of MOG immunoreactivity in the corpus callosum of the different treatment groups was defined as a percentage relative to sections from each group control, which were given the arbitrary value of 100%.

### Evaluation of Myelin Ultrastructure in the Corpus Callosum by Electron Microscopy

Animals used for EM were perfused with PBS followed by cold modified Karnovsky’s fixative (2% paraformaldehyde/2.5% glutaraldehyde). Following perfusion, the brain was removed and postfixed in the same fixative. Coronal sections of the brain (1 mm) were cut between −0.94 and −2.18 of bregma (Franklin and Paxinos, 2008), using a coronal brain slicer (Zivic Instruments, Pittsburg, PA). The corpus callosum was isolated under a dissecting microscope and transferred to 0.1 M cacodylate buffer, postfixed in 1% osmium tetroxide, dehydrated through graded acetone, and embedded in Embed 812 (Electron Microscopy Sciences, Hatfield, PA) using a Pellco Biowave Pro microwave tissue processor (Ted Pella, Inc., Redding, CA). The corpus callosum pieces were oriented for embedding such that semithin sections could be cut at the midline in a sagittal plane. Semithin sections were stained with either toluidine blue, to confirm orientation, or para-phenylenediamine (PPD; MP Biomedicals Inc, Solon, OH) for quantification of myelinated axons (as described later). Ultrathin sections (80 nm) were cut, mounted on copper grids, and stained with uranyl acetate and lead citrate for EM. Electron micrographs were captured on a Technai G2 transmission electron microscope (FEI, Hillsboro, OR).

### Quantification of Myelinated Axons in the Corpus Callosum

Myelinated axons were quantified from 2-µm plastic sections of corpus callosum embedded for EM (as described earlier) and stained with 1% PPD, by the procedure of Hollander and Vaaland (1968). Sections were imaged with a Zeiss Axiomat Imager.2 microscope using a 100 × oil Plan-Apochromat objective (NA 1.3) and photographed with an AxioCamMRm camera and Zeiss Axiovision software (Carl Zeiss Microscopy GmbH, Goettingen, Germany). Three sections, with six areas of 400 µm^2^ per section, were evaluated from two animals for each group (one animal for controls). The myelinated fibers in each area were manually counted using ImageJ software. The number of myelinated fibers (PPD-positive circles) was determined from the six areas for each section. The mean number of PPD-stained axons per area for the different treatment groups was compared with the mean number of axons of each control group.

### Fluorescent Immunohistochemistry and Image Acquisition

For tissue analysis by fluorescent immunohistochemistry, 30-µm free-floating sections were rinsed in PBS and blocked with 5% NDS/0.3% Triton-X 100 for 1 hr at room temperature. All washes were done in PBS. When antigen retrieval was required, sections were transferred to 10 mM sodium citrate, pH 6.0, heated in the Pellco Biowave Pro microwave for 5 min in at 550 W, and rinsed in PBS, prior to blocking. Sections that were labeled with the amyloid precursor protein (APP) antibody were denatured with formic acid prior to blocking. Sections were incubated overnight at 4°C with primary antibodies diluted in 2% NDS. Sections were washed, followed by 1 hr incubation in appropriate secondary antibodies (1:700; Jackson ImmunoResearch) at room temperature. Subsequently, sections were washed, stained with Hoechst (Invitrogen, Carlsbad, CA), mounted on glass slides, and coverslipped with Fluoromount-G (SouthernBiotech, Birmingham, AL). The following antibodies were used for these studies: for oligodendrocyte lineage cells: a rabbit polyclonal to oligodendrocyte transcription factor 2, Olig2 (1:20,000; a gift from Dr. Charles Stiles, Harvard University, Boston, MA); for OPCs: rat anti-platelet-derived growth factor α (PDGFRα,1:500; BD Biosciences, San Diego, CA) or a rabbit polyclonal antibody to chondroitin sulfate proteoglycan, NG2 (1:800; a gift from Dr. William Stallcup, Burnham Institute, La Jolla, CA); for mature myelinating oligodendrocytes: mouse CC1 (1:200; Abcam, Cambridge, MA) or a rabbit antibody to the Pi isoform of glutathione S-transferase pi (GST-pi, 1:500; BD Biosciences); for astrocytes: rabbit anti-glial fibrillary acidic protein (GFAP, 1:600; Sigma-Aldrich, Corp., St. Louis, MO); for microglia: rabbit antibody to ionizing calcium binding adaptor molecule-1 (Iba1, 1:500; WAKO Chemicals USA, Richmond, VA); for proliferating cells: rabbit monoclonal (clone SP6) anti-Ki67 (1:100; Pierce, Thermo Scientific, Rockford, IL); and for axonal damage: rabbit antibody to APP (1:200; Abcam) and a mouse monoclonal antibody to nonphosphorylated neurofilament, SMI32 (Covance Laboratories, Princeton, NJ). Images were acquired with either a Zeiss Axiovert Imager.2 microscope using a 20 × Plan Apochromat (NA 0.8) or 40 × oil Plan Neofluor (NA 1.3) objective and photographed with an AxioCamMRm camera and Zeiss Axiovision software or a Leica SP5 Confocal microscope with LAS software with a 20 × (NA 0.7) objective.

### Oligodendrocyte Quantification

The number of OPCs, mature oligodendrocytes, and proliferating OPCs was quantified for the different treatment groups. To identify OPCs or mature oligodendrocytes in the corpus callosum, free-floating coronal sections (30 µm) were triple labeled with rabbit anti-Olig2, mouse anti-CC1, and rat anti-PDGFRα. All images for these counts were acquired with a Zeiss Axiovert Imager.2 microscope using a 20 × Plan Apochromat (NA 0.8) objective. Image acquisition of the right and left half of the corpus callosum included the area below the midline of the cingulum to the midline of the corpus callosum. The number of Olig2-positive cells (Olig2+, representing all oligodendrocyte lineage cells), CC1+/Olig2+ cells, and PDGFRα+/Olig2+ cells was counted in images from two sections per animal with three animals per control group and four animals in each treatment group at each time point. Cell counts were analyzed to determine the percent of mature (CC1+) and progenitor (PDGFRα+) oligodendrocytes. The percentage was determined by dividing the number of CC1+/Olig2+ or PDGFRα+/Olig2+ cells by the total number of Olig2+ cells in each section. The mean and standard deviation were determined for each group. Representative images in the figures were captured with a Leica SP5 Confocal microscope using a 20 × (NA 0.7) objective. The difference in the number of proliferating OPCs in cuprizone- or cuprizone plus rapamycin-treated mice was determined by double labeling with rabbit Ki67 antibody, which labels all proliferating cells and rat anti-PDGFRα. All images for these counts were acquired with a Zeiss Axiovert Imager.2 microscope using a 40 × oil Plan Neofluor (NA 1.3) objective. Images of the corpus callosum were acquired as described earlier, and the total number of PDGFRα-positive cells and double-positive PDGFRα+/Ki67+ cells was counted. Three animals from each group were evaluated, using two sections from each animal. The mean number and standard deviation were determined for each group.

### APP Quantification

APP was quantified by determining the density of APP-positive objects in confocal images of the corpus callosum using MATLAB (Mathworks, Natick, MA). Confocal images were captured using a 20 × (NA 0.7) objective. Subtracting connected pixels with an area less than 3 or greater than 50 pixels from the image eliminated background levels of fluorescence. Thresholding was applied to the image, and connected objects were quantified. APP density was determined by dividing the number of APP-positive objects/tissue area.

### Statistical Analyses

All statistical analyses were performed using GraphPad Prism software, V 6.0 (GraphPad Software, La Jolla, CA). Data are shown as the mean and standard deviation. In all studies, comparison of mean values was conducted using one-way analysis of variance (ANOVA) analysis with Bonferonni’s correction for multiple comparisons.

## Results

Induction of acute demyelination with cuprizone is typically performed by feeding adult mice a diet of 0.2% to 0.3% cuprizone. Previously, Lindner et al. (2008) demonstrated that a higher degree of demyelination could be achieved with 0.3% cuprizone (w/w) than 0.2%, and the current studies were undertaken using this cuprizone concentration. Treatment groups were fed 0.3% cuprizone mixed into rodent chow and received either vehicle or rapamycin injection. Control groups received either vehicle or rapamycin injection but were fed a regular rodent diet. All groups were analyzed after 4 or 6 weeks of treatment or were returned to a normal diet after 6 weeks treatment and analyzed at different time points thereafter.

### Rapamycin Did Not Increase Cuprizone-Induced Weight Loss

No general weight loss occurred in animals given daily rapamycin injections (Rapa) compared with vehicle-injected mice (Veh; Table 1). The mice on the cuprizone diet (Cup) lost weight, and the addition of rapamycin (CupR) did not significantly alter that weight loss. In both cuprizone-treated groups, mice weighed significantly less than their control group through the first 5 weeks of treatment; however, by the end of the 6-week treatment period, weights were not significantly different from the control.

**Table 1.** Mean weights of mice during treatment period (6 weeks).

Treatment	Weight of mice during treatment						
	*T* = 0	Week 1	Week 2	Week 3	Week 4	Week 5	Week 6
Veh	23.5 ± 1.2	23.56 ± 1.4	24.4 ± 1.3	24.7 ± 1.7	25.3 ± 1.5	26.7 ± 1.3	25.8 ± 2.9
Rapa	23.4 ± 1.5	23.66 ± 1.5	24.2 ± 1.4	24.0 ± 1.3	24.1 ± 1.3	24.4 ± 1.6*	23.8 ± 2.0
Cup	22.8 ± 1.8	20.86 ± 1.4*	21.4 ± 1.7*	21.7 ± 1.8*	22.6 ± 2.1*	22.5 ± 2.4*	23.2 ± 2.4
CupR	23.1 ± 1.9	21.4 ± 1.7*	21.2 ± 1.4*	22.1 ± 1.5*	22.7 ± 1.4*	22.8 ± 1.5*	24.1 ± 1.3

*Note.* The mean weight of the mice on a regular diet and injected with vehicle (Veh, *n* = 25) was compared with mice injected with rapamycin (Rapa, *n* = 31), cuprizone plus vehicle (Cup, *n* = 35), and cuprizone plus rapamycin (CupR, *n* = 35) after each week of treatment. One week following treatment, the weight of Cup- and CupR-treated mice was significantly different than Veh and Rapa, and this difference persisted for 5 weeks of treatment. However, by the end of the treatment period (Week 6), there was no significant difference between any of the groups. With the exception of Week 5, when vehicle-injected mice had a small weight increase, the weight of the Rapa mice did not differ from Veh mice. Data are the mean ± *SD* analyzed by one-way ANOVA.

* *p* < .0001, with Bonferonni’s correction for multiple comparisons.

### Rapamycin Injection Caused Greater Demyelination in Cuprizone-Fed Mice

**Figure 1.**
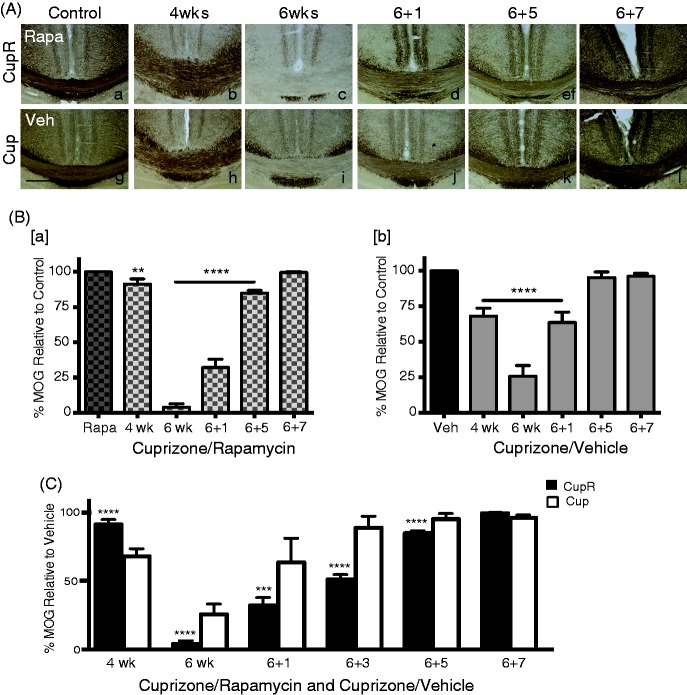
Treatment with cuprizone plus rapamycin caused a greater loss of myelin in the corpus callosum compared with cuprizone alone. (A) Representative coronal sections of the corpus callosum immunostained for MOG from mice treated with regular diet and rapamycin injection (Control, Rapa, a) compared with cuprizone plus rapamycin (upper panels, b to f) or regular diet and vehicle injection (Control, Veh, g) compared with cuprizone plus vehicle (lower panels, h to l) after 4 (b, h) and 6 (c, i) weeks of treatment and recovery times of 1 (d, j), 5 (e, k), and 7 (f, l) weeks. Scale bar = 500 µm. (B) Demyelination and remyelination of the corpus callosum (from the midline to below the cingulum) was quantified by measuring MOG immunoreactivity. Cuprizone plus rapamycin-treated tissue was compared with rapamycin-only controls (B[b]) and cuprizone plus vehicle-treated tissue was compared with vehicle-only controls (B[a]). At each time point, the intensity of MOG immunoreactivity was calculated as the percent of the MOG immunoreactivity in control tissue, which was defined as 100% (see the “Materials and Methods” section). Values are the mean ± *SD* analyzed by ANOVA. **p = .003, ****p < .0001, with Bonferonni’s correction for multiple comparisons. For each time point, *n* = 3–4 animals per treatment. (C) The percent of MOG immunoreactivity in cuprizone and rapamycin tissue (black bars) and cuprizone plus vehicle tissue (white bars) was compared with control levels. ***p < .001, ****p < .0001, with Bonferonni’s correction for multiple comparisons. Scale bar = 500 µm. MOG = myelin oligodendrocyte glycoprotein; Cup = cuprizone alone; CupR = cuprizone plus rapamycin; Rapa = rapamycin alone; Veh = vehicle alone.

Corpus callosum sections were stained for MOG to assess the impact on demyelination with cuprizone or cuprizone plus rapamycin. For simplicity of presentation, the vehicle, rapamycin, cuprizone alone, or cuprizone plus rapamycin treatment groups will be described as Veh mice, Rapa mice, Cup mice, or CupR mice, respectively. Tissue was analyzed from all four groups during the demyelination period at both 4 weeks and 6 weeks of cuprizone exposure. Others have reported that a significant amount of demyelination is present after 4 weeks of cuprizone treatment (Matsushima and Morell, 2001). In agreement, we found that after 4 weeks, significant myelin loss occurred, that is, reduced MOG immunoreactivity relative to control, in both Cup and CupR mice (Figure 1(A)). Interestingly, the initial loss of MOG immunoreactivity was less in the CupR mice (Figure 1(A[b])) than the Cup mice (Figure 1(A[h])). However, by 6 weeks, the reduction in MOG immunoreactivity was greater in CupR mice (Figure 1(A[c])) than Cup mice (Figure 1(A[i])). Treatment with cuprizone plus rapamycin also resulted in greater demyelination in the hippocampus (data not shown). Quantification of MOG immunoreactivity (Figure 1(B)) demonstrated that while MOG immunoreactivity was 25.6% relative to control levels in Cup mice after 6 weeks treatment, MOG immunoreactivity was barely detectable (4%) in CupR mice. The percent of MOG immunoreactivity during demyelination and remyelination under the two treatment conditions was compared with vehicle to better visualize the differences in the two models (Figure 1(C)). The variability among samples was less in the CupR mice than the Cup mice as demonstrated by the smaller error bars.

### Remyelination Developed Slowly Following Removal of Cuprizone Plus Rapamycin and Rapidly Following Removal of Cuprizone

After 6 weeks of cuprizone plus rapamycin or cuprizone, mice were returned to a regular diet for 1, 3, 5, or 7 weeks. As expected, remyelination of the corpus callosum was apparent within the first week of a regular diet, and MOG immunoreactivity continued to increase during the recovery period (Figure 1). Remyelination resulted in an increase of MOG immunoreactivity from approximately 25% of control, to essentially maximal MOG immunoreactivity by 5 weeks of recovery for Cup mice (Figure 1B[b]). In CupR mice, the MOG immunoreactivity was still significantly lower at both 1 and 5 weeks, and 7 weeks of recovery were required to reach control levels (Figure 1B[a]).

**Figure 2.**
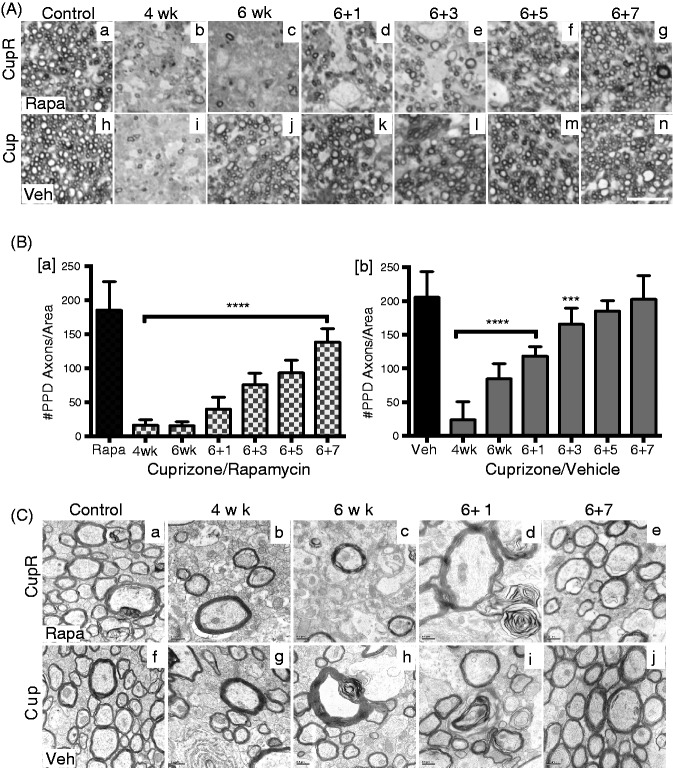
Slower remyelination occurred after treatment with cuprizone plus rapamycin compared with cuprizone alone. (A) Semithin sections (2 µm) were stained with PPD to visualize myelinated axons over a large area of the corpus callosum. Representative areas show control levels of PPD-stained axons in rapamycin-treated (Rapa, a) or vehicle-treated (Veh, h) animals on regular diet compared with the demyelination at 4 and 6 weeks with cuprizone plus rapamycin treatment (b, c) and cuprizone plus vehicle (i, j). The extent of remyelination at 1, 3, 5, and 7 weeks of recovery appeared to be less with CupR (d to g) than with Cup (k to n). Images were captured using a Zeiss 100 × oil Plan-Apochromat objective (NA 1.3). Scale bar = 10 µm. (B[a,b]) Demyelination and remyelination were quantified in PPD-stained sections by manually counting the number of myelinated axons in each 400 µm^2^ area (see the “Materials and Methods” section). Values are the mean ± *SD* analyzed by one-way ANOVA. ***p < .001, ****p < .0001, with Bonferonni’s correction for multiple comparisons. For each time point, *n* = 2 animals per treatment. (C) EM analysis of ultrathin sections was used to visualize the ultrastructure of myelin in the two treatments groups. Representative rapamycin-only treated tissue (a) compared with cuprizone plus rapamycin (top panel) at 4 or 6 weeks (b, c), and recovery times of 1 week (d) and 7 weeks (e). Vehicle-only tissue (f) compared with cuprizone-treated tissue (bottom panel) at 4 weeks (g) or 6 weeks (h) of treatment, and recovery times of 1 week (i) and 7 weeks (j). Scale bar = 0.5 µm. Cup = cuprizone alone; CupR = cuprizone plus rapamycin; Rapa = rapamycin alone; Veh = vehicle alone; PPD = para-phenylenediamine.

Myelinated fibers were quantified in the corpus callosum, to better delineate the difference between these two cuprizone treatment paradigms. Myelinated axons can be visualized in semithin sections stained with PPD, allowing analysis of a larger area of the tissue than can be imaged by EM. Representative PPD-stained sections demonstrated the extent of demyelination and remyelination that occurred both with cuprizone plus rapamycin treatment and cuprizone alone, and during recovery (Figure 2(A)). With either treatment, few myelinated axons were present at 4 weeks (Figure 2(A[b]) and (A[i])). By 6 weeks, only a small number of myelinated fibers were present in CupR mice (Figure 2(A[c])), appearing similar to the 4-week tissue, while in Cup mice, there was a large increase in myelinated fibers (Figure 2(A[j])) compared with 4 weeks. In CupR mice, no major increase in myelinated fibers was detected until 3 to 5 weeks of recovery (Figure 2(A[e]) and (A[f])). Quantification showed that at 4 weeks, the number of myelinated axons was only 10% to 15% of control for both treatments (Figure 2(B)). In Cup mice, the number of myelinated fibers had increased to 41% of control after 6 weeks of treatment (Figure 2(B[b])). This increase in myelinated fibers suggests that the attempt by oligodendrocytes to remyelinate axons in the corpus callosum, which begins sometime after 4 weeks of treatment, did in fact generate robust remyelination despite the presence of cuprizone. In contrast, with the addition of rapamycin, the number of myelinated fibers after 6 weeks remained at 10.6% of control in CupR mice. Thus, the addition of rapamycin to the cuprizone demyelination model appeared to prevent remyelination from occurring during the treatment period. One week after removal of cuprizone, the number of myelinated axons reached ∼60% of control in Cup mice, and by 5 weeks of recovery, myelinated axons were not significantly different from control (Veh; Figure 2(B[b])). Remyelination in CupR mice following removal of cuprizone plus rapamycin had a different pattern (Figure 2(B[a])). After 1 week of recovery, the absence of myelinated axons in CupR mice was striking (21% of control). The number of myelinated axons increased slowly over time, and even after 7 weeks of recovery, although myelinated axons had increased dramatically, the number was significantly lower than control (74.6% of Rapa).

When the ultrastructure of the myelin was analyzed by EM (Figure 2C), the changes in myelinated axons were consistent with the myelinated fibers quantified with PPD staining, indicating a significantly greater loss of myelin in animals treated with cuprizone plus rapamycin than with cuprizone alone. This loss of myelinated axons is demonstrated in Figure 2(C[c]), showing only a few myelinated axons. Remyelination after 7 weeks appeared similar for both groups with respect to the general appearance of the ultrastructure of the myelinated axons (Figure 2(C[e]) and (C[j])), although the PPD data (Figure 2(B)) suggest that full recovery of myelinated axons in CupR mice had not occurred.

### Following Cuprizone Treatment, the Number of OPCs Was Greatly Increased, Whereas the Number of Mature Oligodendrocytes Was Dramatically Decreased

**Figure 3.**
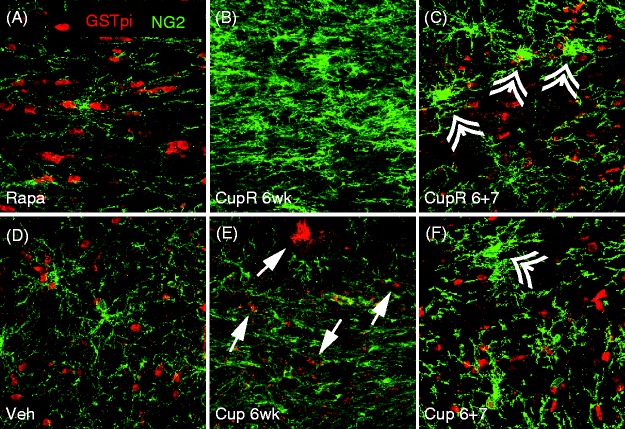
Loss of mature oligodendrocytes and increased OPCs occurred in the corpus callosum following cuprizone plus rapamycin (CupR) and cuprizone (Cup) treatment. Free-floating sections were double labeled for GST-pi and NG2. Confocal images show the presence of mature GST-pi-positive oligodendrocytes (red) in Rapa- (A) or Veh- (D) treated animals. In mice treated with cuprizone plus rapamycin (B), the corpus callosum was completely devoid of GST-pi-positive cells. GST-pi-positive oligodendrocytes decreased substantially after 6 weeks of cuprizone (E), although a small number was still detectable (arrows). By 7 weeks of recovery (6 + 7), GST-pi-positive cells were present in both CupR and Cup tissue (C and F, respectively). In the absence of cuprizone, OPCs, as identified by NG2 immunoreactivity (green), were low in both Rapa and Veh animals (A, D). Following 6 weeks of cuprizone treatment, with either cuprizone plus rapamycin or cuprizone alone, NG2-positive cells increased dramatically. After 7 weeks of recovery, NG2-positive cells were markedly diminished (C, F), although many cells maintained an activated appearance (double arrowheads), with more of these present in CupR tissue (C). Even after 7 weeks of recovery, the numbers of NG2-positive cells appeared greater than control levels. Scale bar = 50 µm. Veh = vehicle alone; Rapa = rapamycin alone; Cup = cuprizone alone; CupR = cuprizone plus rapamycin.

The presence of OPCs and mature oligodendrocytes was assessed in the corpus callosum of mice treated for 6 weeks with cuprizone plus rapamycin or cuprizone alone. Sections were stained for GST-pi, a mature myelinating oligodendrocyte marker (Tansey and Cammer, 1991), and NG2 proteoglycan, an OPC marker (Linthicum et al., 1981; Stallcup, 1981; Figure 3). As expected, a dramatic loss of mature oligodendrocytes was noted in the corpus callosum after 6 weeks of cuprizone treatment in mice of both groups, but the reduction of mature oligodendrocytes was greater in the CupR mice (Figure 3(B)). Arrows in Figure 3(E) show that some remaining GST-pi-positive cells were still present in Cup tissue compared with Figure 3(B), where no GST-pi cells were seen. In both CupR and Cup mice, the number of OPCs expressing NG2 was increased, relative to control, with a greater increase of OPCs in the CupR mice (Figure 3(B)). These changes in oligodendrocytes were clearly in response to the cuprizone diet, as very little difference was seen in the number of OPCs or mature oligodendrocytes between the rapamycin-only-treated mice, compared with vehicle-treated mice (Figure 3(A) and (D), respectively). Recovery was apparent after 7 weeks, with increased numbers of mature oligodendrocytes and reduced numbers of progenitor cells in both the CupR and the Cup groups (Figure 3(C) and (F)), which correlated with the increase seen in MOG immunoreactivity (Figure 1(A[f]), (A[l]), and (B)). Interestingly, NG2-positive cells were larger (double arrowheads, Figure 3(C) and (F)) in CupR and Cup mice compared with controls, and there appeared to be more of these large progenitors in both CupR and Cup mice relative to controls, even at 7 weeks of recovery.

**Figure 4.**
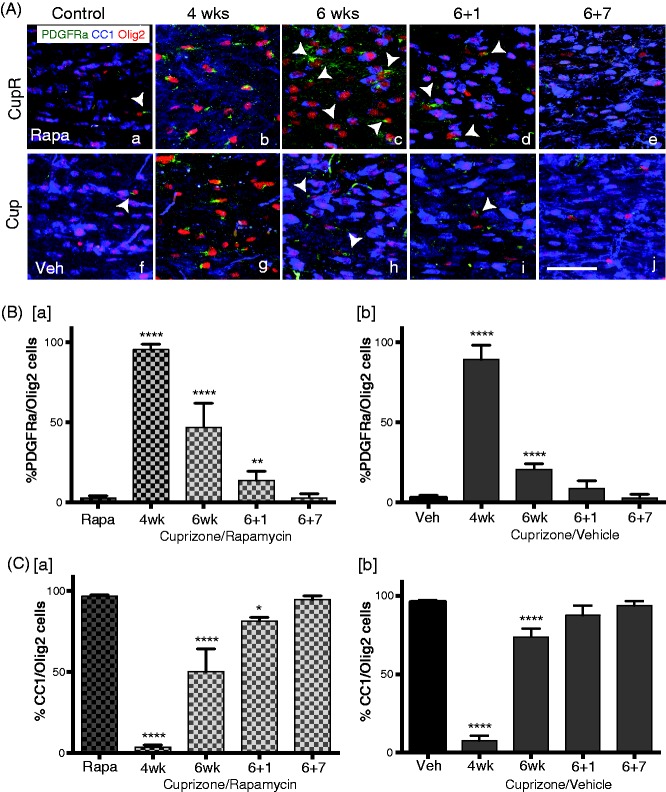
Delayed maturation of oligodendrocyte progenitor cells in the corpus callosum following cuprizone/rapamycin (CupR) as compared with cuprizone (Cup). (A) Free-floating sections were triple labeled with Olig2 (for all oligodendrocyte lineage cells), CC1 (for mature oligodendrocytes), and PDGFRα (for OPCs). Upper panel: representative confocal images from the corpus callosum from Rapa mice (a) and CupR mice (b to e). Lower panel: Veh mice (f) and Cup mice (g to j). Arrowheads indicate some of the PDGFRα/Olig2+ cells. To quantify the changes in oligodendrocytes following treatment, the percentage of all Olig2+ cells that were either PDGFRα+/Olig2+ or CC1+/Olig2+ was determined. (B) Quantification of the percent of OPCs (PDGFRα+/Olig2+) in CupR and Cup tissue for each treatment group at 4 and 6 weeks of treatment and 1 and 7 weeks of recovery, relative to control (Rapa, Veh). (C) The percentage of mature oligodendrocytes (CC1+/Olig2+) in cuprizone plus rapamycin or cuprizone tissue was quantified for each group at 4 and 6 weeks of treatment and 1 and 7 weeks of recovery, relative to the relevant controls (Rapa, Veh). Values are the mean ± *SD* analyzed by one-way ANOVA. **p < .01, ****p < .0001, with Bonferonni’s correction for multiple comparisons. For each time point, *n* = 3–4 animals per treatment. Confocal images were captured using a 20 × (NA 0.7) objective. Scale bar = 50 µm. Rapa = rapamycin alone; CupR = cuprizone plus rapamycin; Veh = vehicle alone; Cup = cuprizone alone; PDGFRα = platelet-derived growth factorα; olig2 = oligodendrocyte transcription factor 2.

To better visualize individual oligodendrocytes for quantification of progenitor cells and mature myelinating oligodendrocytes in the corpus callosum, sections were triple labeled with Olig2/PDGFRα/CC1 (Figure 4). Single OPCs in the corpus callosum were more easily detected with PDGFRα (Figure 4(A)) than with NG2 (Figure 3). GST-pi stains only mature myelinating oligodendrocytes, while CC1 stains both early differentiating oligodendrocytes as well as the more mature myelinating oligodendrocytes (unpublished). In the absence of cuprizone, no differences were seen between the numbers of progenitor (PDGFRα-positive) and maturing (CC1-positive) cells of Rapa animals (Figure 4(A[a])), compared with vehicle-treated mice (Figure 4(A[f])) on a normal diet. However, in animals fed with cuprizone (CupR and Cup), many more PDGFRα+/Olig2+ progenitors and almost no CC1-positive cells (Figure 4(A[b]) and 4([g])) were present after 4 weeks of treatment. Quantification of PDGFRα+/Olig2+ progenitors (Figure 4(B)) confirmed a dramatic increase in the percent of Olig2+ cells that were OPCs after 4 weeks of treatment in both CupR and Cup mice (95.4% ± 3.2 and 89.3% ± 8.94, respectively). After 6 weeks of treatment, the majority of cells in Cup mice was CC1+/Olig2+ mature oligodendrocytes (Figure 4(A[h])), with only a few PDGFRα+/Olig2+ present. In contrast, in the CupR mice, although the number of mature oligodendrocytes had increased, progenitor cells (PDGFRα+/Olig2+) were still present in large numbers (Figure 4(A[c])). Quantification showed that in both groups, the percent of Olig2+ cells that were PDGFRα+ decreased between Week 4 and Week 6 of cuprizone treatment (Figure 4(B)). However, the percent dropped more noticeably with cuprizone alone (Figure 4(B[b]), 20.6% ± 3.43 of Olig2 cells) than with cuprizone plus rapamycin (Figure 4(B[a]), 46.9% ± 14.9 of Olig2 cells). By 7 weeks of recovery in both treatment groups, the number of oligodendrocyte progenitors was reduced to control levels.

We also quantified the number of mature oligodendrocytes (Figure 4(C)) and confirmed that at 4 weeks, only a small percentage of oligodendrocytes in both CupR and Cup mice were CC1+/Olig2+ cells (3.53% ± 3.52 and 7.967% ± 7.32, respectively). The percent of these mature oligodendrocytes increased by 6 weeks with either treatment, when cuprizone was still present in the diet (Figure 4(C)), but the increase was far greater in the Cup mice (Figure 4(C[b])), suggesting that mature oligodendrocytes had differentiated from the high number of progenitors, because at this time, the number of progenitor cells had dropped precipitously in Cup mice. During the recovery period, the percent of CC1+/Olig2+ cells increased in both groups, reaching control levels by 7 weeks.

**Figure 5.**
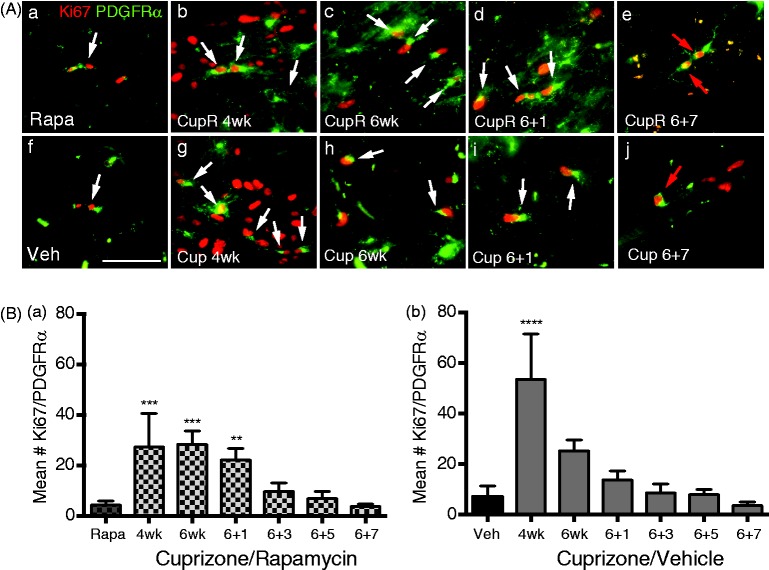
Proliferation of oligodendrocyte progenitors cells (OPCs) in the corpus callosum following cuprizone/vehicle (Cup) or cuprizone/rapamycin (CupR). Proliferating OPCs were labeled with Ki67 (red), a marker of cell proliferation, and PDGFRα (green), a marker of OPCs. Proliferating OPCs were identified as cells that colabeled for both Ki67 and PDGFRα. (A) White arrows indicate PDGFRα+/Ki67+ cells. Upper panel: representative areas of the corpus callosum from rapamycin-treated mice (Control, Rapa, a), compared with mice treated for 4 or 6 weeks with cuprizone plus rapamycin (CupR, b, c) or allowed to recover for 1 or 7 weeks (d, e). Lower panel: representative areas of the corpus callosum from vehicle-treated tissue (Control, Veh, f) and from mice treated for 4 or 6 weeks with cuprizone (Cup, g, h) or allowed to recover for 1 or 7 weeks (i, j). Seven weeks following termination of treatment, a few PDGFRα/Ki67+ cells were still present in CupR and Cup tissue (red arrows, e, j). Images were acquired using a 40 × oil Plan Neofluor (NA 1.3) objective. Scale bar = 50 µm. (B) Quantification of proliferating OPCs at 4 and 6 weeks of treatment and 1, 3, 5, and 7 weeks of recovery. Ki67+/PDGFRα+ cells were counted from images of the right and left half of the corpus callosum, which included the midline of the cingulum to the midline of the corpus callosum with *n* = 3 animals per group. Values are the mean ± *SD* analyzed by one-way ANOVA. **p < .01, ***p < .001, ****p < .0001, with Bonferonni’s correction for multiple comparisons. Rapa = rapamycin alone; CupR = cuprizone plus rapamycin; Veh = vehicle alone; Cup = cuprizone alone; PDGFRα = platelet derived growth factorα.

OPC proliferation was examined by double labeling for Ki67 and PDGFRα (Figure 5). A dramatic increase in Ki67-immunoreactive cells occurred in both groups at 4 weeks (Figure 5(A[b]) and 5(A[g])), and some of these Ki67-positive cells were also PDGFRα-positive (Figure 5(A), arrows). Ki67/PDGFRα double-positive cells appeared to decrease by 6 weeks, while many nonproliferating PDGFRα-positive cells were still present. Only a few Ki67/PDGFRα-positive cells were detectable at 7 week of recovery (red arrows, Figure 5(A[e]) and (A[j])). To determine whether there was a difference in OPC proliferation between the two treatment groups, the number of Ki67-positive cells that were also PDGFRα-positive was quantified (Figure 5(B)). The mean number of Ki67/PDGFRα-positive cells increased after 4 weeks in both the CupR mice (Figure 5(B[a])) and Cup mice (Figure 5(B[b])), with a larger number in mice treated with cuprizone (53.1 ± 18.1) than with cuprizone plus rapamycin (27.3 ± 13.4). After 6 weeks of treatment with cuprizone, the number of Ki67/PDGFRα cells decreased in Cup mice (25.3 ± 4.35). The number of Ki67/PDGFRα-positive cells in the CupR mice at 6 weeks (28.4 ± 5.2) remained comparable with 4 weeks of cuprizone plus rapamycin treatment, and this was also comparable with the number of Ki67/PDGFRα-positive cells in the 6-week Cup mice. After 1 week of recovery, the number of proliferating OPCs was reduced in Cup mice but remained slightly elevated (although not significantly different from control). By contrast, in CupR mice, the number of proliferating OPCs was still significantly higher than control (22.1 ± 4.5 compared with 4.33 ± 1.53). The number of proliferating OPCs continued to fall during the recovery period and approached control levels by 3 weeks of recovery.

### Glial Reactivity Was Greatly Increased Following Cuprizone With or Without Rapamycin Treatment

**Figure 6.**
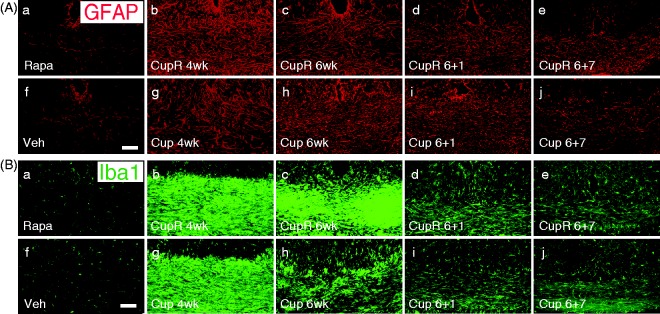
Astrocyte and microglial immunoreactivity increased in the corpus callosum of mice following cuprizone or cuprizone plus rapamycin treatment. The response of glial cells to cuprizone alone and cuprizone plus rapamycin treatment was examined in confocal images of the corpus callosum. (A) CupR samples (b to e) and Cup samples (g to j) were compared with control (Rapa, a; Veh, f) to assess GFAP immunoreactivity in astrocytes. It should be noted that rapamycin alone (Rapa, a) did not induce an astrocytic response. (B) Tissue was stained for Iba1 to assess the microglial response following treatment (a to j). As with the astrocyte response, rapamycin alone (Rapa, a) did not result in activated microglia. Confocal images were captured using a 20 × (NA 0.7) objective. Scale bar = 50 µm. Rapa = rapamycin alone; CupR = cuprizone plus rapamycin; Veh = vehicle alone; Cup = cuprizone alone.

We also examined the effects of the cuprizone or cuprizone plus rapamycin treatment on astrocyte (GFAP) or microglial (Iba1) responses (Figure 6(A) and (B)). In the absence of cuprizone, no difference in glial activation was detected with rapamycin treatment (Rapa, Figure 6(A[a]) and (B[a])) compared with vehicle treatment (Veh; Figure 6(A[f]) and (B[f])). However, significant differences were seen for cuprizone plus rapamycin treatment compared with cuprizone alone. GFAP expression increased at 4 weeks, with stronger reactivity in CupR mice (Figure 6(A[b])) than Cup mice (Figure 6(A[g])), due to either a greater number of cells or denser processes. At 6 weeks, GFAP expression was similar in both CupR and Cup mice, with decreasing levels during recovery. However, GFAP immunoreactivity was still higher than control for both CupR and Cup mice at 7 weeks. Increases in the microglial response, as seen with Iba1 (Figure 6(B)), were even more dramatic than the GFAP response. Iba1 immunoreactivity was elevated in both CupR and Cup tissue at 4 weeks (Figure 6(B[b]) and (B[g])). In Cup mice, the level of Iba1 immunoreactivity decreased by 6 weeks of treatment (Figure 6(B[h])). In contrast, it remained very high in CupR mice at 6 weeks (Figure 6(B[c])). The microglial response subsided during the recovery period, although, as with GFAP, Iba1 immunoreactivity in CupR and Cup mice remained higher than control levels after 7 weeks recovery.

### Axonal Pathology Increased Following Cuprizone With or Without Rapamycin Treatment

**Figure 7.**
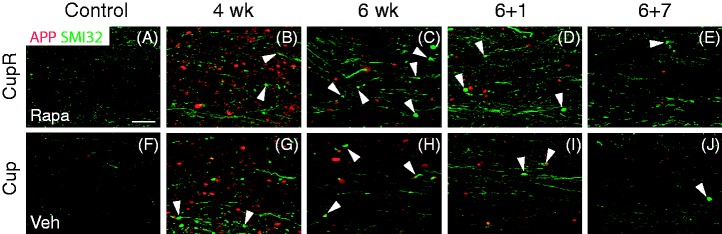
Amyloid precursor protein (APP) and SMI32 increased during cuprizone/rapamycin (CupR) or cuprizone (Cup) treatment. Low levels of APP (red) and SMI32 (green) were present in the corpus callosum from rapamycin-treated (Rapa, A) and vehicle-treated (Veh, F) animals. Increased immunoreactivity for both APP and SMI32 was detectable after 4 weeks with both CupR (B) and Cup (G) mice. Levels of APP decreased at 6 weeks of treatment (C, H) and continued to decline during the recovery period for both CupR (D, E) and Cup (I, J) mice. SMI32 was higher than control levels for both treatments throughout treatment and recovery for both CupR (C–E) and Cup (H–J). White arrowheads identify thick SMI32-immunoreactive spheroids, which are indicative of dystrophic axons. Confocal images were captured using a 20 × (NA 0.7) objective. Scale bar = 25 µm. Rapa = rapamycin alone; CupR = cuprizone plus rapamycin; Veh = vehicle alone; Cup = cuprizone alone.

To examine the effects of cuprizone-induced demyelination on axons in the corpus callosum, we stained with two markers of axonal damage: APP and nonphosphorylated neurofilament (SMI32). Increased APP and SMI32 labeling demonstrated that there was a significant effect on axons in the corpus callosum (Figure 7). Although SMI32 immunoreactivity is normally seen in axons, its accumulation in axonal spheroids is a measure of axonal pathology as is the appearance of APP in axons (Lindner et al., 2009; Xie et al., 2010). In both CupR and Cup mice, APP immunoreactivity increased dramatically in axons after 4 weeks (Figure 7(B) and (G)). Although APP remained high throughout the treatment period, the levels of APP were reduced at 6 weeks in both groups, before cuprizone was removed from the diet (Figure 7(C) and (H)). APP expression declined further as the animals shifted to the remyelination period. Quantification of the density of APP immunoreactivity showed a 42-fold increase in 4-week CupR tissue and 35-fold increase in 4-week Cup tissue (data not shown). Although APP levels dropped significantly at 6 weeks, the density of APP was still greater than control (2.7-fold in CupR mice and 5.5-fold over control in Cup mice). Consistent with high levels of APP, thick SMI32-positive spheroids (white arrowheads) and thick processes were abundant in both treatment groups throughout the demyelination period and at 1 week of recovery. More of these spheroids and thick processes were present in the CupR tissue (Figure 7(C) and (D)) than Cup (Figure 7(H) and (I)); however, the level of SMI32 immunoreactivity was not quantified. The SMI32-positive spheroids were still present but were reduced in both groups at 7 weeks.

## Discussion

The cuprizone-induced model of demyelination has been extensively used to study the process of remyelination and has provided invaluable information regarding the effectiveness of interventions (Torkildsen et al., 2009; Wergeland et al., 2011; Deverman and Patterson, 2012). However, the interpretation of results is confounded by the significant attempt of OPCs to remyelinate tissue during the cuprizone treatment itself (Skripuletz et al., 2011, 2013). In this study, we present data demonstrating that treating mice with a combination of cuprizone and rapamycin results in more complete demyelination and slower remyelination than treatment with cuprizone alone. With respect to the standard cuprizone-induced demyelination model, much of the fundamental data for this study have been described previously (Kipp et al., 2009). Consistent with the degree of demyelination seen in other cuprizone studies (Hibbits et al., 2012), cuprizone treatment without rapamycin for 6 weeks resulted in a 60% reduction in myelin. A greater reduction (90%) resulted from the combination of cuprizone and rapamycin, and more time was required for remyelination in CupR mice. Quantification of MOG immunoreactivity (Figure 1(B)) and PPD myelinated axons (Figure 2(B)) demonstrated the difference in the time course of remyelination between the two treatments. This difference is further emphasized in Figure 1(C), which compares the two treatment groups with vehicle treatment. This suggests that cuprizone plus rapamycin may result in greater demyelination at 6 weeks by preventing underlying remyelination that occurred with cuprizone treatment alone.

The reproducible attempt by OPCs to remyelinate tissue during the cuprizone treatment (Skripuletz et al., 2011, 2013) is in fact a positive observation with respect to remyelination in multiple sclerosis and other human demyelinating diseases. Extensive remyelination occurs in multiple sclerosis, and strong evidence of the attempt to remyelinate is seen in shadow plaques of multiple sclerosis tissue. Nevertheless, this attempt to remyelinate in the human brain is eventually insufficient for full recovery from the multiple sclerosis demyelinating attacks. The degree of remyelination after recovery from cuprizone plus rapamycin indicated that, though there was greater demyelination and axonal pathology, the damage to oligodendrocytes and axons was not permanent. In studies designed to identify remyelination therapeutics, quantifying enhanced remyelination that results from drug treatment rather than baseline endogenous repair is difficult when myelin is still present, as it is in the typical cuprizone model. Therefore, the cuprizone/rapamycin model, which results in nearly complete loss of myelin, may make it easier to identify remyelination therapeutics, as the ability to enhance remyelination can be quantified with a starting point that has less myelin, and remyelination can be measured over a broader time frame.

The addition of rapamycin to the cuprizone treatment was designed to block the underlying remyelination that frequently occurs between Week 3 and Week 6 of cuprizone treatment (Kipp et al., 2009; Skripuletz et al., 2011, 2013) and makes studies to test mechanisms of remyelination and therapeutics difficult. One measure of remyelination is a decrease in OPCs and the concomitant reappearance of mature oligodendrocytes. The greater numbers of NG2- and PDGFRα-labeled OPCs in CupR mice (Figures 3 and 4) illustrate the difference in the two treatments, suggesting the presence of rapamycin was reducing premature remyelination. An increase in OPCs was detected at 4 weeks in both Cup and CupR mice (Figure 4(A) and (B)), and at 6 weeks, the number of OPCs in the CupR mice was higher than Cup mice, presumably because these cells were blocked from further differentiation by exposure to rapamycin. Fewer CC1-positive cells were present in the CupR mice relative to Cup mice (Figure 4(C)), supporting the concept that fewer cells can differentiate to the mature CC1-positive state in the presence of rapamycin.

To combat remyelination insufficiency, studies to identify potential therapeutics that enhance the ability of human OPCs to remyelinate multiple sclerosis lesions are currently underway. The novelty of our study is that the combination of cuprizone and rapamycin, as described here, may provide a highly reproducible methodology for testing remyelination therapeutics. Other modes of rapamycin delivery are also available. Thus, in addition to the labor intensive daily injection protocol used here, it may be possible to delivery rapamycin loaded on nanoparticles (Zou et al., 2011) or micelles (Cho, 2011; Shin et al., 2011; Chen et al., 2013). How effective this delivery to the brain would be and whether it would provide greater or less accurate delivery of rapamycin is unclear at this point.

Although its specific mechanism of action resulting in demyelination is still unclear, cuprizone has been shown to induce a stress response in oligodendrocytes. In particular, Goldberg et al. (2013) showed that even a few days of exposure to cuprizone results in decreases in availability of specific amino acids and an induction of an amino acid response in oligodendrocytes. Such an amino acid response would likely also inhibit mTOR, as mTORC1 is a nutrient-sensing signaling molecule in numerous cell environments (Avruch et al., 2009). The proposed purpose of mTORC1 inhibition is to reduce protein synthesis, thereby protecting the cell during stress. Thus, it would be expected that mTORC1 may be reduced in oligodendrocytes that overexpress ATG3, a component of the amino acid response, in cuprizone animals (Goldberg et al., 2013). In the Goldberg et al. (2013) study, the overexpressing ATF3 cells were also APC-positive, which are classically considered mature oligodendrocytes. The fact that rapamycin, a potent mTOR inhibitor, has a further impact on preventing the underlying remyelination by OPCs, suggests that the stress response induced by cuprizone is only in the mature oligodendrocytes, not in OPCs. Furthermore, it is additional evidence that mTOR activity is involved in the initial differentiation of OPCs to maturing oligodendrocytes as other studies have suggested (Narayanan et al., 2009; Tyler et al., 2009).

Interestingly, OPC proliferation was differentially impacted by cuprizone/rapamycin treatment (Figure 5). Despite the fact that there were similar numbers of OPCs at 4 weeks with both treatments, cuprizone plus rapamycin treatment significantly reduced OPC proliferation. Thus, the concept that rapamycin was blocking differentiation of OPCs may be accurate, but there is likely an additional negative impact of rapamycin on the proliferation of these progenitor cells. This has not been reported before and may be important for studies of remyelination in other contexts. Our observation that rapamycin reduced OPC proliferation is consistent with the study of Paintlia et al. (2013). That study showed that increasing PTEN activity in cultured OPCs, which should decrease Akt/mTOR signaling in a manner similar to rapamycin, also decreased proliferation. Other studies have shown that neural precursor cells that lose mTORC1, the target of rapamycin, have an altered cell cycle, with reduced neural precursor cell proliferation (Cloetta et al., 2013). A more thorough understanding of the regulation of OPC proliferation will be useful for future remyelination studies.

Glial changes have been noted in numerous studies of cuprizone toxicity, and both astrocyte reactivity and microglial activation have been noted (Hiremath et al., 1998; Hibbits et al., 2012; Voss et al., 2012). In the current study, glial reactivity was upregulated early during cuprizone treatment, which is consistent with other studies using the cuprizone model (Hiremath et al., 1998; Hibbits et al., 2012). In Cup mice, the astrocyte response was maximal at 6 weeks as seen previously in the cuprizone model. However, in CupR mice, GFAP immunoreactivity appeared maximal at the 4-week time point, suggesting that the exposure to rapamycin generated a stronger, earlier astrocyte response. In both Cup and CupR mice, GFAP expression was equivalent in both treatment groups at 6 weeks and diminished once treatment ceased. However, it was still higher than control levels after 7 weeks recovery for both Cup and CupR mice. Microglial immunoreactivity was maximal in both Cup and CupR mice after 4 weeks. However, in contrast to the astrocyte response, at 6 weeks, the level of Iba1 immunoreactivity in the CupR mice was still quite elevated, whereas the Iba1 expression in Cup mice had begun to diminish. The degree of microglial activation rapidly decreased once treatment ceased in both groups as seen in other studies using the cuprizone model (Hiremath et al., 1998; Hibbits et al., 2012; Voss et al., 2012), and this decrease continued during the remyelination period.

Despite the known immunosuppressive role of rapamycin, its use can have differential impact on glial activation in rodent models of neurological diseases (Zeng et al., 2008; van Vliet et al., 2012). It can prevent increases in astrocyte reactivity in some epilepsy models (van Vliet et al., 2012) while having little effect on astrocyte reactivity in other epilepsy models (Zeng et al., 2008). In the current studies, rapamycin exposure did not reduce astrocyte reactivity (Figure 6). Furthermore, while in stroke models, rapamycin reduces microglial reactivity (Xie et al., 2014), it is clear that the addition of rapamycin to the cuprizone treatment did not reduce microglial reactivity but actually increased it. Thus, some property of rapamycin enhanced the microglial response during the full 6-week treatment period with cuprizone/rapamycin. However, once treatment ended, microglial reactivity diminished in a manner similar to that in cuprizone alone. Given the fact that there was no reduction of microglia/astrocyte reactivity when rapamycin was included compared with cuprizone alone, the use of rapamycin to reduce premature remyelination likely resulted more from an impact on oligodendrocyte progenitor differentiation than on reduced glial activation. Other studies of corpus callosum demyelination demonstrate increased microglial reactivity during demyelination and decreased reactivity once remyelination begins (Voss et al., 2012). Interestingly, the reduction in Iba1 immunoreactivity in Cup mice at 6 weeks correlates with the increase in myelination that occurs before cuprizone is withdrawn. By contrast, the high level of Iba1 immunoreactivity at 6 weeks in the CupR mice provides further evidence that remyelination does not occur until the treatment with cuprizone plus rapamycin has ceased.

The presence of axonal pathology is a hallmark of multiple sclerosis (Dutta and Trapp, 2007). It has also been demonstrated in the cuprizone model (Lindner et al., 2009; Xie et al., 2010), making it a particularly useful model in which to study neuroprotection and neuroprotective treatments that can be tested during (Yoshikawa et al., 2011) or after (Tsiperson et al., 2010) cuprizone treatment. Yoshikawa et al. (2011) demonstrated that treatment of animals with MK886, a 5-lipoxygenase inhibitor, for the last 7 days of a cuprizone diet significantly reduced the number of APP-positive axons. The cuprizone/rapamycin treatment produced a greater number of APP-positive axons than cuprizone alone. Therefore, this model of demyelination may provide a more sensitive system in which to study the effectiveness of neuroprotective drugs.

Rapamycin inhibition of mTOR not only enhances the demyelination in the cuprizone model, but it also lends insight into the signaling mechanisms through mTOR that regulate remyelination. Two distinct complexes, mTORC1 and mTORC2, are involved in mTOR signaling. Our laboratory has shown that oligodendrocyte-specific loss of Raptor (mTORC1) results in a delay in oligodendrocyte maturation during developmental myelination in the corpus callosum (Bercury et al., 2014), similar to that seen in this study following recovery from cuprizone/rapamycin treatment in adults. Rapamycin-mediated inhibition of mTOR in our demyelination model most likely reduced signaling through Raptor (mTORC1), and reductions of its known target, pS6RP, were seen in mice treated with rapamycin alone as well as cuprizone/rapamycin (data not shown). These data support the role of mTOR, and more specifically mTORC1, as a positive mediator of oligodendrocyte differentiation in the context of remyelination. The other main signaling kinase pathway, mitogen-activated protein kinase (MAPK), which has been shown to play a critical role in regulating active myelination *in vivo* during development (Ishii et al., 2013), also has a similar role in remyelination in the model of lysolecithin-induced demyelination (Fyffe-Maricich et al., 2013). These data support conserved roles of the protein kinases in modulating oligodendrocyte differentiation in both development and remyelination. Disruption of either of these signaling pathways may result in the failure of efficient remyelination in diseases such as multiple sclerosis and provide insight into potential targets that could enhance myelin production.

## Summary

This study describes a novel cuprizone demyelination model that results in greater demyelination than the standard cuprizone model. This model is useful for analyzing mechanisms of remyelination in the adult rodent nervous system and for testing therapeutics to enhance remyelination.

## Authors' Note

Drs. Popescu and Dr. Narayanan initially developed the cuprizone/rapamycin demyelination model, designing and carrying out extensive preliminary studies. Ms. Sachs and Dr. Bercury generated the data and edited the manuscript. Dr. Macklin directed the study and edited the manuscript.
